# Barriers to care in juvenile localized and systemic scleroderma: an exploratory survey study of caregivers’ perspectives

**DOI:** 10.1186/s12969-023-00819-6

**Published:** 2023-04-25

**Authors:** Leigh A. Stubbs, Andrew M. Ferry, Danielle Guffey, Christina Loccke, Erin Moriarty Wade, Pamela Pour, Kaveh Ardalan, Peter Chira, Ingrid M. Ganske, Daniel Glaser, Gloria Higgins, Nadia Luca, Katharine F. Moore, Vidya Sivaraman, Katie Stewart, Natalia Vasquez-Canizares, Raegan D. Hunt, Renata S. Maricevich, Kathryn S. Torok, Suzanne C. Li, Childhood Arthritis, Rheumatology Research Alliance (CARRA) Scleroderma Workgroup

**Affiliations:** 1 Department of Pediatrics, Division of Rheumatology, Baylor College of Medicine and Texas Children’s Hospital, Houston, TX USA; 2 Department of Plastic Surgery, Division of Pediatric Plastic Surgery, Baylor College of Medicine and Texas Children’s Hospital, Houston, TX USA; 3 Institute for Clinical and Translational Research, Baylor College of Medicine, Houston, TX USA; 4 Board of Directors, National Scleroderma Foundation, Danvers, MA USA; 5 Boca Raton, FL USA; 6 Advisory Board Member, Scleroderma Foundation Michigan Chapter, Southfield, MI USA; 7 Department of Pediatrics, Division of Rheumatology, Duke University School of Medicine, Durham, NC USA; 8 Department of Pediatrics, Division of Rheumatology, University of California San Diego, San Diego, CA USA; 9 Department of Plastic and Oral Surgery, Boston Children’s Hospital and Harvard Medical School, Boston, MA USA; 10 Department of Pediatrics, Section of General Pediatrics, Yale University, New Haven, CT USA; 11 Department of Pediatrics, Division of Rheumatology, Ohio State University College of Medicine and Nationwide Children’s Hospital, Columbus, OH USA; 12 Department of Pediatrics, Division of Pediatric Rheumatology, University of Calgary and Alberta Children’s Hospital Research Institute, Calgary, AB Canada; 13 Department of Pediatrics, Division of Pediatric Rheumatology, University of Colorado School of Medicine, Aurora, CO USA; 14 Department of Pediatrics, Division of Pediatric Rheumatology, University of Texas Southwestern, Dallas, TX USA; 15 Department of Pediatrics, Division of Pediatric Rheumatology, Albert Einstein College of Medicine and Children’s Hospital at Montefiore, Bronx, NY USA; 16 Department of Dermatology, Baylor College of Medicine and Texas Children’s Hospital, Houston, TX USA; 17 Department of Pediatrics, Division of Pediatric Rheumatology, University of Pittsburgh and Children’s Hospital of Pittsburgh, Pittsburgh, PA USA; 18 Department of Pediatrics, Division of Pediatric Rheumatology, Joseph M. Sanzari Children’s Hospital, Hackensack Meridian School of Medicine, 30 Prospect Avenue, Hackensack, NJ 07601 USA

**Keywords:** Localized scleroderma, Morphea, Systemic scleroderma, Health care disparities

## Abstract

**Background:**

Juvenile localized scleroderma (LS) and systemic sclerosis (SSc) are rare pediatric conditions often associated with severe morbidities. Delays in diagnosis are common, increasing the risk for permanent damage and worse outcomes. This study explored caregiver perspectives on barriers they encountered while navigating diagnosis and care for their child’s scleroderma.

**Methods:**

In this cross-sectional study, caregivers of juvenile LS or SSc patients were recruited from a virtual family scleroderma educational conference and a juvenile scleroderma online interest group. The survey queried respondents about their child’s condition and factors affecting diagnosis and treatment.

**Results:**

The response rate was 61% (73/120), with 38 parents of LS patients and 31 parents of SSc patients. Most patients were female (80%) and over half were non-Hispanic white (55%). Most families had at least one person with a college education or higher (87%), traveled ≤ 2 h to see their rheumatologist (83%), and had private insurance (75%). Almost half had an annual household income ≥ $100,000 (46%). Families identified the following factors as barriers to care: lack of knowledge about scleroderma in the medical community, finding reliable information about pediatric scleroderma, long wait times/distances for a rheumatology/specialist appointment, balance of school/work and child’s healthcare needs, medication side effects, and identifying effective medications. The barrier most identified as a major problem was the lack of knowledge about juvenile scleroderma in the medical community. Public insurance, household income less than $100,000, and Hispanic ethnicity were associated with specific barriers to care. Lower socioeconomic status was associated with longer travel times to see the rheumatologist/specialist. Diagnosis and systemic treatment initiation occurred at greater than one year from initial presentation for approximately 28% and 36% of patients, respectively. Families of LS patients were commonly given erroneous information about the disease, including on the need and importance of treating active disease with systemic immunosuppressants in patients with deep tissue or rapidly progressive disease.

**Conclusion:**

Caregivers of children with LS or SSc reported numerous common barriers to the diagnosis, treatment, and ongoing care of juvenile scleroderma. The major problem highlighted was the lack of knowledge of scleroderma within the general medical community. Given that most of the caregiver respondents to the survey had relatively high socioeconomic status, additional studies are needed to reach a broader audience, including caregivers with limited English proficiency, geographical limitations, and financial constraints, to determine if the identified problems are generalizable. Identifying key care barriers will help direct efforts to address needs, reduce disparities in care, and improve patient outcomes.

**Supplementary Information:**

The online version contains supplementary material available at 10.1186/s12969-023-00819-6.

## Background

Juvenile scleroderma includes localized scleroderma (LS) and systemic sclerosis (SSc). Although LS and SSc have some common pathophysiology, they present in different patterns with unique morbidities and outcomes. In juvenile LS, extracutaneous involvement is common with morbidities including extremity length discrepancies, joint contractures, seizures, and facial hemiatrophy [1, 2]. SSc is associated with a higher prevalence of multi-organ morbidity, including life-threatening pulmonary, cardiac, renal, and vascular involvement. Delays in diagnosis are common for both LS and SSc, with reported mean delays of 1.2–1.6 and 1.9–2.8 years, respectively [3]. Treatment delays in LS have been associated with more persistent disease activity, higher damage scores, and higher relapse rates [4, 5]. Before methotrexate was recognized as effective treatment, many juvenile LS patients developed severe functional impairment from musculoskeletal morbidity. Studies from that time reported over 40% of juvenile LS patients seen in orthopedic clinics underwent surgical procedures, which were often multiple and included amputations in 5% of patients [5]. For SSc, treatment delays can increase the severity of organ involvement, with juvenile SSc patients in the Childhood Arthritis and Rheumatology Research Alliance (CARRA) Legacy Registry found to have the worst level of functional impact and disability of all the major pediatric rheumatic diseases [6]. There is a need to increase the early diagnosis and treatment of these conditions to improve patient outcomes [5].

Delays in diagnosis and treatment are multifactorial including a condition's prevalence, the familiarity of providers with the disease and its clinical manifestations, referral patterns, and access to care. For LS, there is an incidence of approximately 3 cases per 100,000 children per year [7]. The onset is often insidious with initial symptoms that may mimic bruising, café au lait spots, or other childhood dermatologic disorders. In contrast to other pediatric rheumatology conditions, patients with LS are often referred to a dermatologist prior to a rheumatologist. Within the CARRA registry cohort, most children (94%) already had disease damage features at their first rheumatology visit [2]. The incidence for SSc is even lower at approximately 0.3–0.5 per million children per year [8, 9]. Common features of juvenile SSc include Raynaud’s phenomenon, cutaneous changes, arthralgia, myalgia, gastroesophageal reflux, and failure to thrive. Patients are often referred to several specialists before seeing a rheumatologist and obtaining a diagnosis. Healthcare disparities have been found to affect many health outcomes including disease severity, long-term outcome, and mortality [10–12]. Although access to care and diagnostic delay for several pediatric rheumatic diseases has been studied, there is limited information for juvenile LS and SSc [12–17].

This study aimed to explore the caregiver’s perspective on barriers to accessing specialty care and systemic treatment for juvenile scleroderma. This information could then guide future efforts to develop effective interventions to reduce diagnostic and treatment delays.

## Methods

### Survey design and administration

An electronic survey was designed by the CARRA Scleroderma Workgroup members based on literature review and expert opinion to be distributed to caregivers. The term caregiver was used for inclusivity for any adult with legal custody of a child. The survey consisted of questions in multiple choice, checkbox, and free-response formats. Questions were designed to elucidate the demographic and clinical characteristics of the affected child and the household's demographic characteristics. Additionally, caregivers were queried to estimate the impact of various barriers to care on the diagnosis and treatment of systemic and localized scleroderma. The Barriers to Care Scale (BACS) was adapted based on existing scales and prior pediatric rheumatology barriers to care studies [14–16, 18–21]. Nineteen potential barriers to accessing care were assessed: language, appearance/ancestry/accent, specialist referral, access to specialist, specialist wait time, travel time/distance, transportation, community knowledge of scleroderma, insurance coverage of medication, insurance coverage of specialist appointments, cost of medication/care, caregiver missing work, child missing school, childcare coverage, understanding medication administration, side effects of medications, identification of effective medication, obtaining medication, and reliable information on juvenile scleroderma. Barriers were measured using a four-point Likert scale (1 = “No problem at all,” 2 = “Very slight problem,” 3 = “Somewhat of a problem,” 4 = “Major problem”) to indicate the degree of the barrier to scleroderma care. Lastly, one optional, free-response question was included at the end of the survey. The survey was revised based on feedback from physicians with juvenile scleroderma expertise and caregivers of children with juvenile scleroderma. Four caregivers of children with scleroderma (two systemic and two localized) piloted the survey. The survey is included as an additional file (see Additional file 1).

Surveys were distributed to caregivers of children with LS or SSc via email using REDCap^©^ (Vanderbilt University, Nashville, TN). Caregiver emails used to administer surveys were collected using two different methods. Method one entailed reaching out to caregivers during the free and virtual National Scleroderma Foundation pediatric scleroderma conference (Kids Get Scleroderma Too) held on October 23, 2021. Method two involved caregivers providing their email through an opt-in link posted on the largest scleroderma parent interest group on Facebook “Parents of Scleroderma Kids” (Meta Platforms Inc., Menlo Park, CA) from October through December 2021. This group was a convenience sample since only those at the conference or active in the Facebook group during this time could participate in the survey. Survey participation was optional and restricted to one response per email. No compensation was provided for participation or survey completion. All responses were collected anonymously. This study received ethics board approval from Baylor College of Medicine and Texas Children’s Hospital.

### Statistical analysis

Responses were summarized using descriptive statistics. Frequency with percentage, mean with standard deviation, and median with 25th and 75th percentiles were employed when applicable. Characteristics are compared by type with t-test or ANOVA for normally distributed continuous characteristics, Fisher's exact test for categorical characteristics. Unadjusted logistic regression for the delay to diagnosis and delay to medications assessed the association with patient characteristics. Based on previous access to care pediatric rheumatology research, time to diagnosis was treated categorically with the following groupings: less than one month, between one and three months, between three and six months, between six and twelve months, between one and two years, between two and four years, and more than four years [15, 16]. Time to systemic treatment was treated categorically with the following groupings: less than three months, between three and six months, between six and twelve months, between one and two years, between two and four years, and more than four years. All analyses were performed using Microsoft Excel or Stata v.15.

## Results

### Patient and family characteristics

Seventy-three caregivers (62 mothers, 6 fathers, and one grandmother) responded to the survey (response rate 61%). Families unsure if their child had juvenile scleroderma were excluded from statistical analysis (*n* = 4). Table 1 presents the summary statistics for the patient and family characteristics. The mean age at diagnosis was 8.7 years (SD 3.9). Most patients were female (80%) and over half were non-Hispanic white (55%). Approximately 43% of patients identified as a racial or ethnic minority group including Hispanic white (20%), non-Hispanic black (9%), Asian (9%), Hispanic black (4%), and Hispanic Native American (1%). Most families had at least one person with a college education or higher (87%), had private insurance (75%), and identified English as their primary language (86%). Almost half had an annual household income ≥ $100,000 (46%).

**Table 1:** Patient and caregiver characteristics

	**Total (*n* = 69)**	**Localized (*n* = 38)**	**Systemic (*n* = 31)**
	**N (%/SD)**	**N (%/SD)**	**N (%/SD)**
**On a scale of 0–100 (0 = no impact, 100 = very large impact), how much did the child’s disease have on…**			
Child’s life at diagnosis (mean)	59.2 (31.7)	62.1 (33.5)	55.7 (29.5)
Child’s life currently (mean)	54.4 (27.8)	52.8 (27.7)	56.2 (28.4)
Family’s life overall (mean)	70.5 (27.2)	68.7 (29.2)	72.7 (24.8)
**Mean age of child at diagnosis (years)**	8.7 (3.9)	7.9 (3.8)	9.6 (3.6)
**Mean age of child currently (years)**	12.6 (4.1)	12.2 (4.4)	13.0 (3.6)
**Biological sex of child**			
Female	55 (79.7%)	29 (76.3%)	26 (83.9%)
Male	14 (20.3%)	9 (23.7%)	5 (16.1%)
**Race/ethnicity of child**			
Asian, non-Hispanic	6 (8.7%)	5 (13.2%)	1 (3.2%)
Black, Hispanic	3 (4.3%)	3 (7.9%)	0
Black, non-Hispanic	6 (8.7%)	1 (2.6%)	5 (16.1%)
Native American, Hispanic	1 (1.4%)	1 (2.6%)	0
White, Hispanic	14 (20.3%)	8 (21.1%)	6 (19.4%)
White, non-Hispanic	38 (55.1%)	20 (52.6%)	18 (58.1%)
Prefer not to say	1 (1.4%)	0	1 (3.2%)
**Primary language**			
English	59 (85.5%)	32 (84.2%)	27 (87.1%)
Spanish	5 (7.2%)	3 (7.9%)	2 (6.5%)
Other	5 (7.2%)	3 (7.9%)	2 (6.5%)
**Highest level of education in home**			
Completed a graduate school program	29 (42.0%)	16 (42.1%)	13 (41.9%)
Completed College or university	31 (44.9%)	18 (47.4%)	13 (41.9%)
Completed High School or have a GED	5 (7.2%)	2 (5.3%)	3 (9.7%)
Completed elementary or middle school	2 (2.9%)	1 (2.6%)	1 (3.2%)
Prefer not to say	2 (2.9%)	1 (2.6%)	1 (3.2%)
**Type of insurance**			
Public	15 (21.7%)	6 (15.8%)	9 (29.0%)
Private	52 (75.4%)	30 (78.9%)	22 (71.0%)
None	2 (2.9%)	2 (5.3%)	0
**Annual household income**			
Over $150,000	21 (30.4%)	14 (36.8%)	7 (22.6%)
$100,000- $150,000	11 (15.9%)	6 (15.8%)	5 (16.1%)
$75,000- $99,999	7 (10.1%)	4 (10.5%)	3 (9.7%)
$50,000- $74,999	9 (13.0%)	4 (10.5%)	5 (16.1%)
$25,000- $49,999	9 (13.0%)	5 (13.2%)	4 (12.9%)
Less than $25,000	2 (2.9%)	1 (2.6%)	1 (3.2%)
Unknown or prefer not to say	10 (14.5%)	4 (10.5%)	6 (19.4%)
**US census regions**			
Northeast	19 (27.5%)	12 (31.6%)	7 (22.6%)
Midwest	11 (15.9%)	6 (15.8%)	5 (16.1%)
South	19 (27.5%)	12 (31.6%)	7 (22.6%)
West	10 (14.5%)	5 (13.2%)	5 (16.1%)
Outside of US	10 (14.5%)	3 (7.9%)	7 (22.6%)
**Time to travel to doctor**			
Greater than 5 h	5 (7.2%)	3 (7.9%)	2 (6.5%)
4- 5 h	3 (4.3%)	2 (5.3%)	1 (3.2%)
3- 4 h	1 (1.4%)	1 (2.6%)	0
2- 3 h	3 (4.3%)	3 (7.9%)	0
1- 2 h	18 (26.1%)	9 (23.7%)	9 (29.0%)
Less than 1 h	39 (56.5%)	20 (52.6%)	19 (61.3%)

The geographical representation was mainly within the United States (US) with more families from the Northeast (28%) and South (28%) than the Midwest (16%) and West (15%). There were also caregiver respondents from outside of the US, especially for the SSc group (23%), including Canada (*n* = 2), Spain (*n* = 2), United Kingdom (*n* = 2), Croatia (*n* = 1), New Zealand (*n* = 1), Russia (*n* = 1), and Zimbabwe (*n* = 1). Most families (83%) traveled ≤ 2 h to see their doctor. Out of the 12 families who traveled greater than 2 h, the majority were situated in the Southern US (*n* = 4) and Midwestern US (*n* = 4).

The LS group (*n* = 38) included the following subtypes: circumscribed (*n* = 2), linear (head: *n* = 10, trunk/limbs: *n* = 7), generalized (*n* = 4), mixed (*n* = 12), pansclerotic (*n* = 2), and unclassified (*n* = 1). The SSc group (*n* = 31) included the following subtypes: diffuse cutaneous (*n* = 14), limited cutaneous (*n* = 4), sine scleroderma (*n* = 1), overlap (*n* = 8), and caregivers unsure of subtype (*n* = 4). There were no significant differences between the LS and SSc patient and family cohort characteristics. There were wide standard deviations for the following means: impact on the child’s life at diagnosis (59.2, SD 31.7), child’s life currently (54.4, SD 27.8), and family life overall (70.5, SD 27.2).

### Barriers to accessing care

**Fig. 1:**
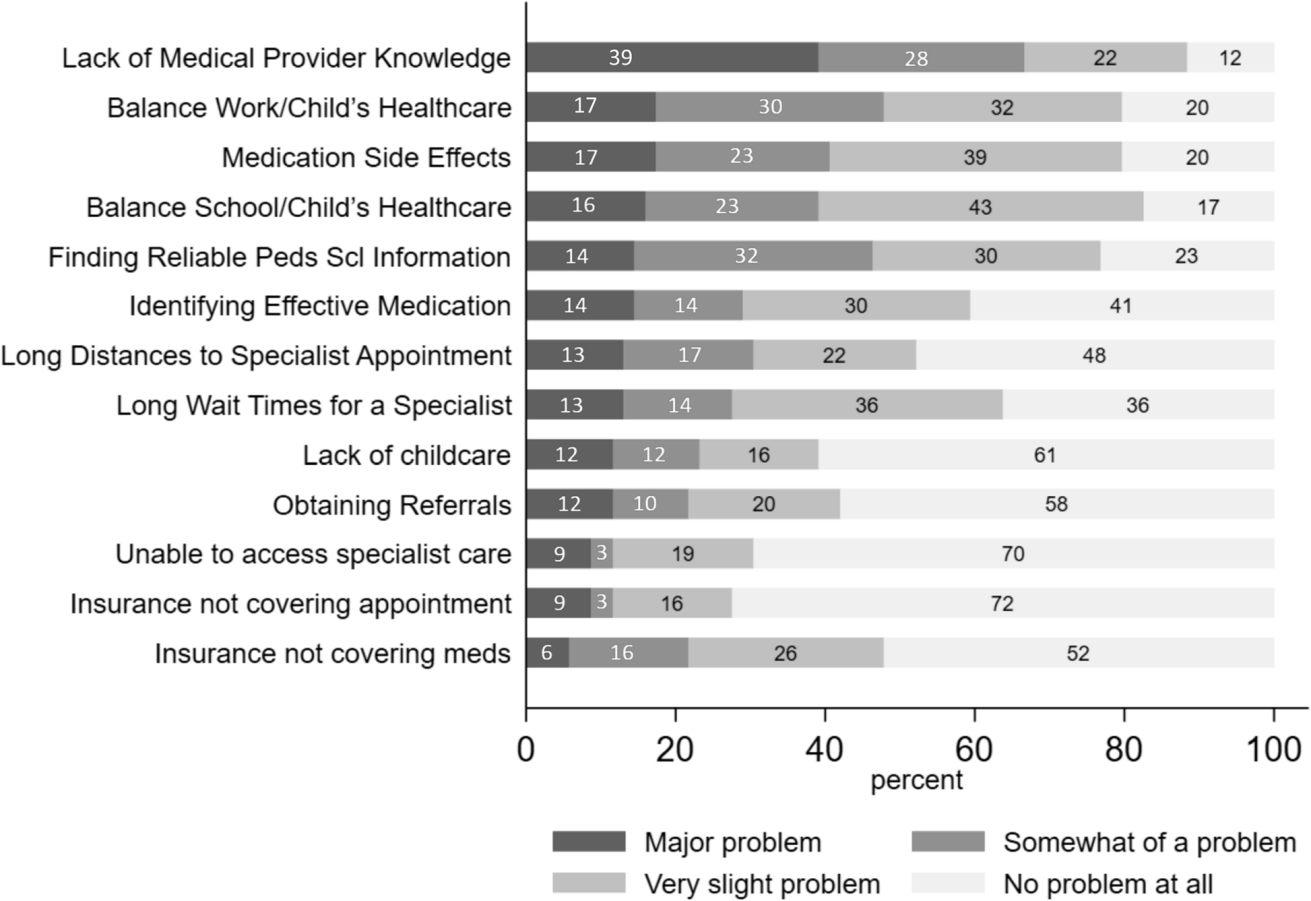
Barriers to accessing care in juvenile scleroderma patients

Figure 1 provides the percentages of the 13 individual barrier items that were perceived as being a major problem by at least 5% of the respondents. Given the small sample size, the primary analyses were conducted with pooled juvenile LS and SSc data. The barrier most frequently identified as a major problem was lack of medical provider knowledge about scleroderma, with 39% rating this a major problem, and only 12% rating this as not a problem. Finding reliable information about pediatric scleroderma was also rated as a problem (slight, somewhat, or major) by most (77%) of respondents, with 14% rating this a major problem, and 32% somewhat of a problem. Other commonly rated barriers were balance of work and child’s healthcare needs (80%), side effects from medications (80%), balance of school and child’s healthcare needs (83%), identifying the medications that improved their child’s symptoms (59%), long distances to specialist appointment (52%), and long wait times for a rheumatology/specialty appointment (64%).

**Fig. 2:**
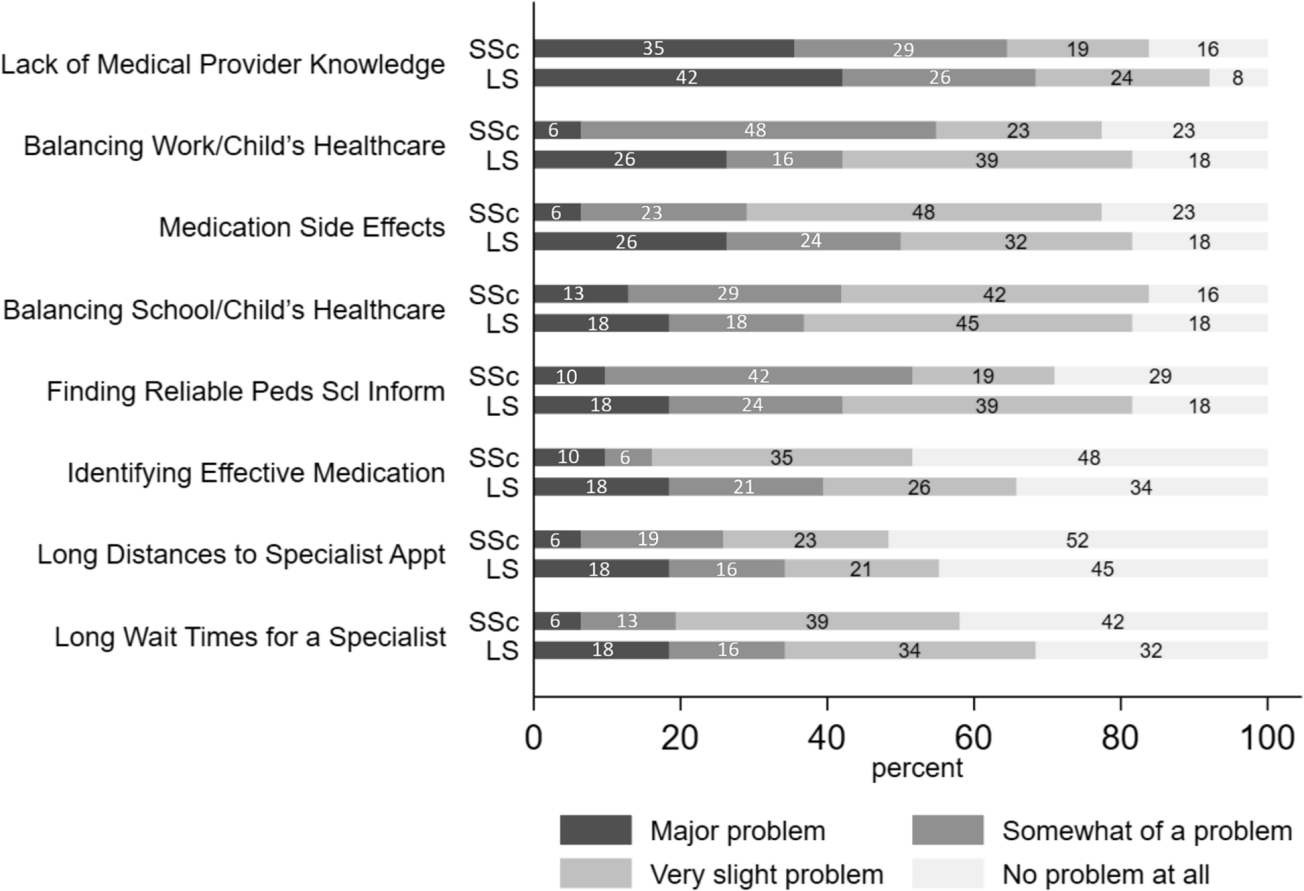
Barriers to care comparison between juvenile localized scleroderma and systemic sclerosis groups

Figure 2 shows the differences between the LS and SSc groups for the eight barriers identified as problems by more than 50% of caregivers. All eight barriers were identified more frequently as a major problem within the LS group than within the SSc group. The highest percent differences between LS and SSc groups were in the medication side effect and balancing work and child’s healthcare needs categories, which were approximately 20% higher in the LS group. However, this exploratory study was not powered to detect differences between LS and SSc, and no significant differences were identified for problems between LS and SSc groups via Fischer’s exact test.

For participants with the US, the associations between the major barriers and social determinants of health were assessed via chi squared analysis (Table 2). Public insurance was associated with household income less than $50,000 (*p* < 0.001), finding doctors fluent in their language (*p* = 0.033), and obtaining specialist referrals (*p* = 0.008). Household income less than $100,000 was associated with travel time over two hours (*p* = 0.005), long distances to appointments (*p* = 0.040), household education of high school or less (*p* < 0.001), and lacking transportation (*p* = 0.003). Hispanic ethnicity was associated with long specialist wait times (*p* = 0.006), affording medications/appointments (*p* = 0.025), and identifying effective medications (*p* < 0.001).

**Table 2:** *P*-values Determined by Chi Squared Analysis for Social Determinants of Health Associated with Barriers^a^ to Care for United States Participants

**Social Determinant**	Public Insurance (*n* = 11)	Income (< $100,000) (*n* = 21)	Hispanic Ethnicity (*n* = 17)
**Income < $50,000**	**< 0.001**	NA	NS
**Travel time > 2 h**	**NS**	**0.005**	NS
**Household education of high school or less**	**NS**	**< 0.001**	NS
**Major problem finding doctors fluent in their language**	**0.033**	NS	NS
**Major problem obtaining referrals for specialist**	**0.008**	NS	NS
**Major problem with long wait times for specialist**	NS	NS	**0.006**
**Major problem accessing a specialists**	0.093	NS	NS
**Major problem with long distances to appointment**	NS	**0.040**	NS
**Major problem with lacking transportation**	NS	**0.003**	NS
**Major problem affording medication or appointments**	NS	NS	**0.025**
**Major problem balancing school and healthcare needs**	0.079	NS	NS
**Major problem identifying effective medication**	NS	0.065	**< 0.001**

^a^Characteristics assessed included public insurance, income, travel time, race, ethnicity, English as primary language, household education, United States regions, doctors fluent in your language, judgement of appearance, obtaining referrals, long wait times for a specialist, long distances to medical appointment, lacking transportation, ability to access specialist, lack of medical provider knowledge of scleroderma, insurance coverage of medications, insurance coverage of appointments, ability to afford medications, balancing work/school and healthcare needs, finding childcare, understanding instructions of medications, side effects from medications, identifying effective medication, obtaining medication from pharmacy, and finding reliable scleroderma information. Only *p*-values < 0.1 were included

### Diagnosis and treatment analysis

Forty-five percent and 42% patients with LS and SSc presented to multiple providers prior to diagnosis, respectively (Table 3). Among LS patients, 21% were only seen by a primary care provider while 32% were only seen by a dermatologist prior to diagnosis. SSc patients were often evaluated only by a primary care provider (48%) or a dentist (10%) prior to diagnosis. LS patients were diagnosed primarily by a dermatologist (63%), whereas SSc patients were diagnosed mainly by a rheumatologist (74%). Among the dermatologists who diagnosed LS patients, approximately 46% were adult dermatologists.

**Table 3:** Diagnostic and treatment cohort characteristics

	**Total (*n* = 69) N (%)**	**Localized (*n* = 38) N (%)**	**Systemic (*n* = 31) N (%)**
**Healthcare provider(s) seen before diagnosis**			
Primary care provider only	23 (33.3%)	8 (21.1%)	15 (48.4%)
Dentist only	4 (5.8%)	1 (2.6%)	3 (9.7%)
Dermatology only	12 (17.4%)	12 (31.6%)	0
Multiple providers including primary care, dermatology, dentist, and other specialists	30 (43.5%)	17 (44.7%)	13 (41.9%)
**Healthcare provider who made diagnosis**			
Primary Care Provider	6 (8.7%)	2 (5.3%)	4 (12.9%)
Dermatology	24 (34.8%)	24 (63.2%)	0
Rheumatology	35 (50.7%)	12 (31.6%)	23 (74.2%)
Other	4 (5.8%)	0	4 (12.9%)
**How much time passed between bringing your child to medical attention and a diagnosis?**			
Less than 1 month	14 (20.2%)	5 (13.2%)	9 (29.0%)
Between 1 and 3 months	16 (23.2%)	9 (23.7%)	7 (22.6%)
Between 3 and 6 months	10 (14.5%)	5 (13.2%)	5 (16.1%)
Between 6 and 12 months	10 (14.5%)	6 (15.8%)	4 (12.9%)
Between 1 and 2 years	9 (13.0%)	8 (21.1%)	1 (3.2%)
Between 2 and 4 years	5 (7.2%)	2 (5.3%)	3 (9.7%)
More than 4 years	5 (7.2%)	3 (7.9%)	2 (6.5%)
**How much time passed between initial symptoms and initiation of systemic medication(s)?**			
Less than 3 months	16 (23.2%)	9 (23.7%)	7 (22.6%)
Between 3 and 6 months	15 (21.7%)	6 (15.8%)	9 (29.0%)
Between 6 and 12 months	9 (13.0%)	4 (10.5%)	5 (16.1%)
Between 1 and 2 years	10 (14.5%)	8 (21.1%)	2 (6.5%)
Between 2 and 4 years	11 (15.9%)	6 (15.8%)	5 (16.1%)
More than 4 years	4 (5.8%)	2 (5.3%)	2 (6.5%)
Not applicable	4 (5.8%)	3 (7.9%)	1 (3.2%)

Approximately 28% of juvenile scleroderma patients were diagnosed after more than a year of symptoms. This occurred more commonly for LS patients (34%) than SSc patients (19%). Unadjusted logistical regression did not show any statistically significant associations with patient characteristics and delay of diagnosis. Within the cohort, 65 patients (94%) were started on systemic treatment with 36% having symptoms greater than one year before treatment. Again, this occurred more commonly for LS patients (42%) than SSc patients (29%).

Families of LS and SSc patients commented that there was a “lack of awareness” within the medical community. Mainly LS patients heard the following myths about their child’s scleroderma: “it will naturally go away or burn out” (*n* = 17), “it is a harmless/cosmetic condition” (*n* = 13), “there is no need to treat with medications” (*n* = 8), and “medications are dangerous or harmful” (*n* = 5). These findings were reinforced in the free-response comments where families described misdiagnosis leading to delayed treatment and the need for multiple medical opinions before a final diagnosis. One family reported being “accused of Munchausen.” Caregivers commented that their child’s LS was misdiagnosed as “eczema,” “allergy,” “hemihypertrophy,” and “vitiligo.” Even when the correct diagnosis was made by non-rheumatology providers, several families commented on having been counseled that the disease would “burn out”, so no treatment was indicated.

Regarding surgical referral, only one of the seven LS patients with a linear subtype affecting a limb was referred to orthopedics, and only four of the ten craniofacial LS patients were referred to plastic surgery.

## Discussion

Both juvenile LS and juvenile SSc are commonly associated with severe morbidities, with 27% of juvenile LS and up to 74% of juvenile SSc patients found to have functional limitations in a North American cohort [2, 6]. In addition, juvenile SSc patients are at risk for developing life-threatening internal organ involvement. In this study, we identified diagnostic and treatment delays of more than one year for approximately three out of ten juvenile scleroderma patients. In contrast, in this same cohort, diagnostic and treatment delays of more than one year were identified for 14.6% of juvenile dermatomyositis and 9% of childhood systemic lupus erythematosus patients [15, 16]. Within our cohort, the diagnostic and treatment delays, as well as barriers identified as major problems, were more frequent within the LS than the SSc group. However, our study was not powered to identify significant differences between the groups.

Within this study, the barriers to juvenile scleroderma care can be divided into interconnected factors of access, medical knowledge, and family resources. Access includes wait times, distance, and referrals. Medical knowledge includes the awareness of juvenile scleroderma among healthcare providers, availability of information resources, and identification of effective medications with minimization of side effects. Family resources include healthcare disparity topics such as language, judgement on appearance/accent, transportation, education attainment, insurance coverage, ability to afford medical costs, and the balance of childcare and healthcare. Some of these barriers will need tailored interventions specific to juvenile scleroderma, whereas others can more broadly apply to pediatric rheumatology, chronic conditions, and rare diseases.

Pediatric rheumatology access is influenced by multiple factors such as pediatric rheumatology workforce supply, demand, and geographic distribution. In 2015, the pediatric rheumatology workforce was estimated to be 300 full-time equivalent providers in the US, or about three providers per million children [22]. By 2030, the projected provider demand will be approximately twice the supply [22]. Furthermore, the pediatric rheumatology workforce is often concentrated in large metropolitan academic centers, and there is an imbalance of geographical distribution throughout the US. There are currently 14 US states lacking a practicing pediatric rheumatologist [22].

In our cohort, there was a broad distribution of patients within the US. The families who travelled the furthest were more often in the Southern and Midwest states. Many caregivers identified long distances (52%) and wait times (64%) as problems. Families with lower socioeconomic status (SES) had statistically significant associations with travel time over two hours, long distances to appointments, and lacking transportation. Long distances to a pediatric rheumatologist can increase diagnostic delays and alter pediatrician referral patterns for rheumatic diseases [12, 15, 16, 20]. Pediatricians may manage the patient independently or refer to other specialists [22]. Ongoing research and interventions working to address pediatric rheumatology workforce issues include boosting physician and nonphysician recruitment, increasing workforce diversity, extending telemedicine use, and creating incentive programs to redistribute providers to underserved areas [12, 19, 22].

Given that the barrier most classified as a major problem was the lack of knowledge about juvenile LS and SSc in the medical community, there is a need for increased awareness of these rare diseases. Juvenile scleroderma patients were often seen by multiple providers prior to diagnosis, but the pattern of providers differed between LS and SSc. Juvenile LS patients were often diagnosed by dermatologists, with nearly half being diagnosed by adult dermatologists. Unlike LS, SSc patients were often seen by primary care providers and dentists before the diagnosis was established by a pediatric rheumatologist. A prior United Kingdom study also noted similar juvenile LS and SSc referral patterns and diagnostic delay [17].

The majority of dermatologists in North America, both pediatric and adult, use topical rather than systemic immunosuppressive medicines to treat juvenile LS (morphea), as shown in two surveys of dermatologists [23, 24]. This is very different from the treatment pattern of pediatric rheumatologists, where a survey in North America found 95% use systemic immunosuppressants to treat juvenile LS survey [25]. Recommendations from two major pediatric rheumatology groups favor methotrexate treatment [26, 27]. Some of the difference in treatment patterns between dermatologists and rheumatologists likely reflects differences in disease pattern between adult-onset and juvenile onset LS. In adults, the most common LS subtype is superficial circumscribed morphea, which is usually primarily a relatively short-lived cosmetic concern that is readily controlled by topical treatment. In contrast, linear scleroderma (head and trunk/limb involvement) is the most common pediatric subtype, and this subtype is typically associated with deep tissue involvement resulting in serious morbidities such as arthropathy, muscle atrophy, limb length difference, facial hemiatrophy, seizures, uveitis, and dental defects [5, 27]. In general, pediatric onset LS is associated with a high frequency of extracutaneous involvement, long disease duration (mean 13–14 years), high relapse rate, and high potential to impair permanent growth unlike adult-onset LS [5, 28]. Therefore, the management of most juvenile LS patients differs significantly from that for most adult-onset LS patients. For juvenile LS, early initiation of systemic immunosuppressive treatment in patients with deep tissue or rapidly progressive active disease, continual screening for extracutaneous involvement, and long-term monitoring for persistence or reoccurrence of disease activity is essential [5, 28, 29]. The lack of awareness of these differences in disease patterns, treatment needs, and outcomes between pediatric versus adult onset disease, can lead to inappropriate under treatment and a missed opportunity to limit the severity of extracutaneous morbidity, resulting in higher disease burden and poorer outcome in juvenile LS patients.

The LS families were more likely to encounter a lack of awareness of the potential severity and morbidities associated with LS. They were often erroneously told that there was no need to treat their child, the condition was harmless, and medications were more dangerous than the disease. These myths reflect the understanding of LS treatment prior to studies establishing the effectiveness of methotrexate [5]. A 1977 juvenile LS review stated the most important aspect of treatment was vigorous occupational and physical therapy, and the lack of effective medical therapies was reiterated in a 1996 review that stated, “Linear scleroderma does not respond well to any treatment, although many therapies have been tried. … No treatment has been shown to work consistently in the disease” [30, 31]. Another paper in 2000 stated that the, “treatment of scleroderma, medically, has been largely unsuccessful. …treatment with a number of drugs including anti-inflammatory and immunosuppressive agents has not proven consistently effective” [32]. Methotrexate began to be used in LS following a 1996 double blinded, placebo controlled, randomized clinical trial (DB PC RCT) showing its effectiveness for skin thickening in systemic sclerosis [33]. A DB PC RCT in juvenile LS demonstrated the effectiveness of methotrexate for controlling active disease in 2011 [34], and a 2019 Cochrane review favors treating active disease in juvenile LS with methotrexate plus prednisone [35].

Strategies to increase medical provider awareness of juvenile scleroderma need to involve primary care, dermatology, orthopedic, plastic surgery, and dental providers. Role-play simulation and patient educators have previously improved recognition of SSc [36]. By focusing on teaching “red flags,” workshops could be applied to both forms of juvenile scleroderma as well as other pediatric rheumatology conditions. Moreover, provider and family knowledge can be enhanced through partnerships with physician and disease foundations for information resources.

Both physicians and caregivers acknowledge the challenges of identification of effective treatment and medication side effects for juvenile scleroderma. Caregivers in this study reported identifying effective medications (nearly 60%) and side effects (80%) as problems. For most juvenile LS subtypes, methotrexate remains the first-line therapy [27–29]. Adjunctive corticosteroid recommendations vary [26, 27]; two of the CARRA-generated consensus treatment plan (CTP) regimens include corticosteroids, either intravenous or oral administration [26]. One variant of the CTP with intravenous corticosteroids, recommends this be given weekly for 12 weeks, a regimen that could create challenges for balancing healthcare and work/school needs [26]. Unfortunately, approximately one-third of patients fail methotrexate therapy, which likely contributes to the higher percent of medication side effects noted in the LS group [34, 37]. Within the CARRA pilot comparative CTP study, all the patients who experienced methotrexate failure had extracutaneous manifestations, and extracutaneous involvement was identified to be associated with longer persistence of disease activity and use of more treatment and longer treatment durations in another prospective study [37, 38]. Other treatments include mycophenolate mofetil, tocilizumab, abatacept, infliximab, and JAK inhibitors for refractory juvenile LS [28, 29]. For juvenile SSc, the Single Hub and Access point for pediatric Rheumatology in Europe (SHARE) recently published the first consensus-based recommendations in 2020 [39]. In general, juvenile SSc treatment is guided by organ involvement and is mostly adapted from adult studies because of the paucity of jSSc treatment studies. For example, early recognition of “silent” interstitial lung disease (ILD) by high-resolution computed tomography of the lungs allows for treatment to prevent or impede the progression of permanent organ damage [40]. Early diagnosis is essential since ILD is the leading cause of morbidity and death for children and adults with SSc [40]. Ongoing research is being done to assess potential reliable clinical, biomarker, imaging, or laboratory features to identify patients at risk for a refractory course and optimize therapy outcomes.

Although guidelines and CTPs can help to standardize care, the understanding of healthcare disparities is essential to improve the equity of juvenile scleroderma care. Like many survey studies, there may be selection bias since only families who attended the virtual family scleroderma educational conference or were a part of the online support group were surveyed. Overall, most of the respondents were of relatively high socioeconomic status and had private insurance. Given their participation in support groups and educational conferences, these families likely have more resources and time to devote to understanding scleroderma compared to many other families with a child with juvenile scleroderma. Nonetheless, these caregivers still identified many difficulties navigating care for their children, and a substantial impact of the disease upon their child’s and family’s life. We were able to identify greater barriers in US families on public insurance, with household incomes < $100,000, and of Hispanic ethnicity. It seems likely that this survey underestimates disease-related problems among the families of all patients with juvenile scleroderma. Due to the exploratory nature of the study, it was also not powered to detect differences between the different subgroups, and type II errors may exist. In addition, there were only eight families from a variety of countries other than North America, certainly insufficient to discern differences in disparities within the international scleroderma community. Future studies may improve generalizability and increase sample size by offering questions in different languages, and by conducting surveys in a wide variety of clinics as well as during telemedicine appointments.

Additional studies could assess correlation of accumulated social disadvantage (low guardian education, low household income level, underinsured status, and high adverse childhood experience score) with juvenile LS and SSc severity [13].

## Conclusions

This is the first study to evaluate barriers to care in both forms of pediatric scleroderma and identify areas of need. The strengths of this study include correlations to previous studies and focusing on caregiver stakeholders. Compared to the CARRA Legacy Registry, the age of onset and disease subtypes were similar for both LS and SSc, but notably the cohort in the current study was more racially diverse. Similar delays in initiation of treatment for scleroderma in children were found in our current study and in the CARRA registry [2, 6]. Prior studies on social determinants of health in pediatric rheumatology have focused on administrative datasets and CARRA Registry data. In contrast, this study uniquely focused on the caregiver perspective, which helps to assess the root cause of disparities at a family level. We were able to identify the lack of knowledge a common barrier to care for both LS and SSc, with LS families more likely to encounter myths about the importance and need for systemic immunosuppressive treatment. We identified greater barriers in families that were located further away from pediatric rheumatologists and had lower socioeconomic status. The combination of registries and caregiver studies will help to guide the development of individual, institution, and system-level advocacy leading to improved recognition and treatment of juvenile localized scleroderma.

## Supplementary Information

Additional file 1. Scleroderma Barriers to Care Survey.

## Data Availability

The data supporting this study's findings are available on request from the corresponding author. The data are not publicly available due to privacy and ethical restrictions.

## Abbreviations

CARRA: Childhood arthritis and rheumatology research alliance
CTP: Consensus treatment plan
ILD: Interstitial lung disease
LS: Localized scleroderma
SHARE: Single hub and access point for pediatric rheumatology in Europe
SSc: Systemic scleroderma
US: United States
