# Supplementary material for: Barriers to care in juvenile localized and systemic scleroderma: an exploratory survey study of caregivers’ perspectives
Source: Pediatr Rheumatol Online J. 2023 Apr 25;21:39. doi: 10.1186/s12969-023-00819-6 (PMC10131373; doi:10.1186/s12969-023-00819-6)
Supplement: Supplementary file 1 — Additional file 1. Scleroderma Barriers to Care Survey. [file 12969_2023_819_MOESM1_ESM.pdf]

Scleroderma Barriers to Care Survey

Please complete the survey below.

Dear Families,

We are exploring the barriers or challenges you may have encountered while seeking care for your child with localized or systemic scleroderma. We are looking to assess factors that may have led to a delay in diagnosis or treatment.

We would greatly appreciate 5-10 minutes of your time to complete a brief survey. Would you please answer these questions as they relate to your child with scleroderma? Please have only one person from your family complete the survey once, even if you have more than one child with scleroderma.

All responses will be collected anonymously through REDCap. If you decide to take part in this study, this will serve as your consent. You may stop the survey at any time. There is no compensation for your participation in this study. We hope that the results will help medical providers and patients in the future. Any publications or presentations of the research results will be discussed as grouped data rather than individual data.

By clicking the yes button and providing a response, you consent to participate in this study. Please contact Dr. Suzanne Li, suzanne.li@hmhn.org, or Dr. Leigh Anna Stubbs, leigh.stubbs@bcm.edu, should you have any questions or concerns regarding the study. Additionally, you may withdraw from the study at any time using the same contact information. Thank you very much for your time and participation.

Sincerely,

Suzanne Li, MD/PhD
Professor of Pediatric Rheumatology, Hackensack Meridian School of Medicine

Leigh Anna Stubbs, MD/MPH
Pediatric Rheumatology Fellow, Baylor College of Medicine

○ Yes
○ No

Which type of scleroderma does your child have?

○ Systemic sclerosis/scleroderma: limited or extensive with involvement of the skin, and often internal organs (esophagus, gut, lungs, kidneys, and/or heart)
○ Localized scleroderma/morphea: primarily involving skin and often nearby tissues such as joints and muscles. Other names include linear scleroderma, en coup de sabre, and Parry-Romberg Syndrome.
○ Both systemic sclerosis and localized scleroderma
○ I do not understand the difference between localized and systemic scleroderma
○ Unsure whether my child has scleroderma

At DIAGNOSIS, on a scale 0-100, please give a number to rate the impact your child's disease had on his/her life? (0 = no impact, 100 = very large impact)	No impact ... Very large impact<br>(Place a mark on the scale above)
At this CURRENT time, on a scale 0-100, please give a number to rate the impact your child's disease has on his/her life? (0 = no impact, 100 = very large impact)	No impact ... Very large impact<br>(Place a mark on the scale above)
OVERALL, on a scale 0-100, please give a number to rate the impact your child's disease has had on the ENTIRE family? (0 = no impact, 100 = very large impact)	No impact ... Very large impact<br>(Place a mark on the scale above)
What is your relationship to the child with scleroderma?	◯ Father<br>◯ Mother<br>◯ Non-parental Legal Guardian<br>◯ Other
Please specify:	______________________
Do other people (non-guardians) help care for your children (e.g., babysitting, transportation, etc.)?	◯ Yes<br>◯ No
How old was your child when they were diagnosed with scleroderma?	______________________<br>(In Years)
What is your child's current age?	______________________<br>(In Years)
What is your child's biological sex?	◯ Female<br>◯ Male<br>◯ Prefer not to say
Is your child of Hispanic, Latinx, or Spanish origin?	◯ No, not of Hispanic, Latinx, or Spanish origin<br>◯ Yes, Mexican, Mexican American, Chicano<br>◯ Yes, Puerto Rican<br>◯ Yes, Cuban<br>◯ Yes, another Hispanic, Latinx, or Spanish origin<br>◯ Prefer not to say
What is your child's race? Click all that apply.	☐ African American or Black<br>☐ American Indian or Alaskan Native<br>☐ Asian<br>☐ White<br>☐ Other<br>☐ Prefer not to say
Please specify:	______________________

What is the primary language spoken at home?	◯ English<br>◯ Spanish<br>◯ Other
Please specify:	______________________________
What is the highest level of education completed in your household?	◯ Completed elementary or middle school<br>◯ Completed High School or have a GED<br>◯ Completed College or university (2-year or 4-year)<br>◯ Completed a graduate school program (e.g., Master's degree, PhD, MD, DDS, JD)<br>◯ Prefer not to say
Does your child have health insurance?	◯ Yes<br>◯ No
What type of insurance do they have?	◯ Private<br>◯ Public (Medicaid, CHIP, Other Governmental Insurance)<br>◯ Other
Please specify:	______________________________
What was the annual income range of your household, from all sources (in US $), for the last year?	◯ Less than $25,000<br>◯ $25,000- $49,999<br>◯ $50,000- $74,999<br>◯ $75,000- $99,999<br>◯ $100,000- $150,000<br>◯ Over $150,000<br>◯ Unknown or prefer not to say
What U.S. state do you live in? If not in the U.S., then list the country that you live in.	______________________________
How long does it take you to travel to see the doctor who treats your child's scleroderma?	◯ Less than 1 hour<br>◯ 1- 2 hours<br>◯ 2- 3 hours<br>◯ 3- 4 hours<br>◯ 4- 5 hours<br>◯ Greater than 5 hours<br>◯ Unsure

How much of a problem were the following for your family when obtaining care for your child's scleroderma?

	No problem at all	Very slight problem	Somewhat of a problem	Major problem
Doctors were not fluent in your language.	◯	◯	◯	◯

Being judged on your appearance, your ancestry, or your accent.	○	○	○	○
Obtaining referrals for rheumatology or other specialist. (e.g., dermatology, plastic surgery)	○	○	○	○
Long wait times for a rheumatology or specialist appointment.	○	○	○	○
Long distances to medical facilities and providers.	○	○	○	○
Lacking transportation to access the services I need.	○	○	○	○
Unable to access to a pediatric rheumatologist or pediatric specialist.	○	○	○	○
Lack of knowledge about scleroderma in the community.	○	○	○	○
Insurance not covering medications.	○	○	○	○
Insurance not covering appointments for scleroderma specialists.	○	○	○	○
Unable to afford medications or medical appointments.	○	○	○	○
Balancing work and child's healthcare needs.	○	○	○	○
Balancing school and child's healthcare needs.	○	○	○	○
Hard to find others to care for my other children or other family members in my household.	○	○	○	○
Understanding the instructions on how to take the medications.	○	○	○	○
Side effects from the medications.	○	○	○	○
Identifying the medication(s) that improved my child's symptoms.	○	○	○	○
Issues obtaining the medications from the pharmacy.	○	○	○	○
Finding reliable information about pediatric scleroderma.	○	○	○	○

What type(s) of localized scleroderma/morphea does your child have?	◯ One oval/circular flat lesion on body<br>◯ One sunken or depressed lesion on body (an indented or scooped out appearance of the skin)<br>◯ Multiple lesions<br>◯ Linear (band-like) lesion on face or scalp (e.g., en coup de sabre, Parry-Romberg Syndrome)<br>◯ Linear (band-like) lesion on body<br>◯ Lesion on all sides of a limb (circumferential) that may have affected the underlying tissues, such as fascia, muscle, and/or bone (pansclerotic morphea)<br>◯ Mixed (combination of the different types described above)<br>◯ Unsure
Which part of your child's body was FIRST affected by scleroderma?	◯ Face and/or scalp<br>◯ Neck, Chest, and/or Breast<br>◯ Abdomen<br>◯ Back<br>◯ Hip and/or Buttock<br>◯ Arm and/or hand<br>◯ Leg and/or foot<br>◯ Other/Multiple Sites
Please specify:	__________________________________
What were the FIRST symptoms or signs of your child's scleroderma?	☐ Changes in skin color<br>☐ Changes in skin texture or feel (harder, waxy, thinner, smoother, tighter)<br>☐ Changes in sensation at site of skin lesion (itchy, tingling, pulling, pain, or other odd sensation)<br>☐ Hair loss<br>☐ Problem with joint(s): harder to move, pain with movement, limited mobility, swollen or tender<br>☐ Feeling pain or weakness in muscles<br>☐ Uneven growth: difference in size between 2 sides of body or head<br>☐ Other/Multiple Symptoms
Please specify:	__________________________________
Which health care provider(s) did you see for these symptoms BEFORE your child was diagnosed with scleroderma? Click all that apply	☐ Dermatologist (skin doctor): Adult/general<br>☐ Dermatologist (skin doctor): Pediatric<br>☐ Dentist/orthodontist<br>☐ Emergency or urgent care doctor<br>☐ Family medicine doctor (general doctor for children and adults)<br>☐ Homeopath/Naturopath/Holistic practitioner/Healer<br>☐ Ophthalmologist (eye doctor)<br>☐ Orthopedic doctor (bone doctor)<br>☐ Pediatrician (general doctor for children)<br>☐ Physician assistant or nurse practitioner<br>☐ Plastic surgeon<br>☐ Rheumatologist: Adult<br>☐ Rheumatologist: Pediatric<br>☐ No one<br>☐ Other

Please specify:	______________________________
Who FIRST DIAGNOSED your child with scleroderma?	◯ Dermatologist (skin doctor): Adult/general<br>◯ Dermatologist (skin doctor): Pediatric<br>◯ Dentist/orthodontist<br>◯ Emergency or urgent care doctor<br>◯ Family medicine doctor (general doctor for children and adults)<br>◯ Homeopath/Naturopath/Holistic practitioner/Healer<br>◯ Ophthalmologist (eye doctor)<br>◯ Orthopedic doctor (bone doctor)<br>◯ Pediatrician (general doctor for children)<br>◯ Physician assistant or nurse practitioner<br>◯ Plastic surgeon<br>◯ Rheumatologist: Adult<br>◯ Rheumatologist: Pediatric<br>◯ Other
Please specify:	______________________________
Who CURRENTLY treats your child's scleroderma? Click all that apply	☐ Adult medicine doctor (internist)<br>☐ Dermatologist (skin doctor): Adult/general<br>☐ Dermatologist (skin doctor): Pediatric<br>☐ Family practice doctor (general doctor for children and adults)<br>☐ Pediatrician (general doctor for children)<br>☐ Plastic Surgeon<br>☐ Rheumatologist: Adult<br>☐ Rheumatologist: Pediatric<br>☐ Other
Please specify:	______________________________
How much time passed between bringing your child to medical attention for these symptoms and receiving a diagnosis of localized scleroderma?	◯ 1 month or less<br>◯ Between 1 and 3 months<br>◯ Between 3 and 6 months<br>◯ Between 6 and 12 months<br>◯ Between 1 and 2 years<br>◯ Between 2 and 4 years<br>◯ More than 4 years
While seeking care for scleroderma, did you hear any of the following from a medical provider? Click all that apply	☐ It is a harmless/cosmetic condition.<br>☐ It will naturally go away or burn out.<br>☐ No need for medications.<br>☐ Physical and occupational therapy are the main treatments.<br>☐ Medications are dangerous or harmful.<br>☐ Children do not get scleroderma.<br>☐ No, I was never told any of the above.<br>☐ Other
Please Specify:	______________________________

For localized scleroderma, has a healthcare provider recommended treatment with methotrexate, mycophenolate mofetil (Cellcept), steroids, and/or other oral, injection, or intravenous (IV) medicines?	◯ Yes, my child was treated with one of these medications<br>◯ Yes, but we declined treatment with these medications<br>◯ No<br>◯ Unsure<br>◯ Prefer not to say
How much time passed between your child's first symptoms and starting systemic treatment (e.g., steroids, methotrexate, immunoglobulin, etc.)?	◯ Less than 3 months<br>◯ 3-6 months<br>◯ 6- 12 months<br>◯ 12-18 months<br>◯ 18-24 months<br>◯ 2-3 years<br>◯ 3-4 years<br>◯ More than 4 years
What made you decide to treat your child's scleroderma with medicine? Click all that apply	☐ My child's primary care provider agreed with this treatment.<br>☐ I received information from the Scleroderma Foundation about this treatment.<br>☐ I talked to another family whose child has scleroderma about the treatment.<br>☐ I trusted the doctor who advised this treatment.<br>☐ My child's disease was worsening.<br>☐ Their current treatment was not working (please list what treatment they were on in "other").<br>☐ Other
Please list any other reason you decided to go ahead with this treatment:	__________________________________
What made you decide to decline medicines for your child's scleroderma? Click all that apply	☐ I was worried about the side effects and safety of these medicines.<br>☐ I preferred to try natural treatments such as herbs, diet, or lifestyle changes.<br>☐ I cannot afford the recommended treatments/medicines.<br>☐ My child's scleroderma was mild.<br>☐ Other
Please specify:	__________________________________
What type of systemic scleroderma does your child have?	◯ Diffuse<br>◯ Limited<br>◯ Overlap (has features of dermatomyositis, lupus, and/or arthritis)<br>◯ Without skin involvement, only affecting the organs inside the body<br>◯ Not sure
What were the FIRST symptoms or signs of your child's systemic scleroderma? Click all that apply.	☐ Cold fingers or fingers/toes changing color<br>☐ Joint pain, swelling, limitation<br>☐ Skin changes: dryer, harder, shinier, swollen, tighter, smoother<br>☐ Muscle Weakness<br>☐ Poor weight gain or weight loss<br>☐ Tired, limited playing<br>☐ Other

Please specify: ______________________________

Which healthcare provider(s) did you see BEFORE your child was diagnosed with systemic scleroderma? Click all that apply.

- ☐ Cardiologist (heart doctor)
- ☐ Dermatologist (skin doctor): Adult/general
- ☐ Dermatologist (skin doctor): Pediatric
- ☐ Dentist/orthodontist
- ☐ Emergency or urgent care doctor
- ☐ Family medicine doctor (general doctor for children and adults)
- ☐ Gastroenterologist/GI (gut doctor)
- ☐ Homeopath/Naturopath/Holistic practitioner/Healer
- ☐ Ophthalmologist (eye doctor)
- ☐ Orthopedic doctor (bone doctor)
- ☐ Pediatrician (general doctor for children)
- ☐ Physician assistant or nurse practitioner
- ☐ Pulmonologist (lung doctor)
- ☐ Rheumatologist: Adult
- ☐ Rheumatologist: Pediatric
- ☐ No one
- ☐ Other

Please specify: ______________________________

Who FIRST DIAGNOSED your child with systemic scleroderma?

- ◯ Cardiologist (heart doctor)
- ◯ Dermatologist (skin doctor): Adult/general
- ◯ Dermatologist (skin doctor): Pediatric
- ◯ Dentist/orthodontist
- ◯ Emergency or urgent care doctor
- ◯ Family medicine doctor (general doctor for children and adults)
- ◯ Gastroenterologist/GI (gut doctor)
- ◯ Homeopath/Naturopath/Holistic practitioner/Healer
- ◯ Ophthalmologist (eye doctor)
- ◯ Orthopedic doctor (bone doctor)
- ◯ Pediatrician (general doctor for children)
- ◯ Physician assistant or nurse practitioner
- ◯ Pulmonologist (lung doctor)
- ◯ Rheumatologist: Adult
- ◯ Rheumatologist: Pediatric
- ◯ Other

Please specify: ______________________________

Who CURRENTLY treats your child's systemic scleroderma? Click all that apply

- ☐ Adult medicine doctor (internist)
- ☐ Dermatologist (skin doctor): Adult/general
- ☐ Dermatologist (skin doctor): Pediatric
- ☐ Cardiologist (heart doctor)
- ☐ Family practice doctor (general doctor for children and adults)
- ☐ Gastroenterologist/GI (gut doctor)
- ☐ Pediatrician (general doctor for children)
- ☐ Pulmonologist (lung doctor)
- ☐ Rheumatologist: Adult
- ☐ Rheumatologist: Pediatric
- ☐ Other

Please specify: ______________________________

How much time passed between bringing your child to medical attention for these symptoms and receiving a diagnosis of systemic scleroderma?	◯ 1 month or less<br>◯ Between 1 and 3 months<br>◯ Between 3 and 6 months<br>◯ Between 6 and 12 months<br>◯ Between 1 and 2 years<br>◯ Between 2 and 4 years<br>◯ More than 4 years
While seeking care for scleroderma, did you hear any of the following from a medical provider? Click all that apply	☐ It is a harmless/cosmetic condition.<br>☐ It will naturally go away or burn out.<br>☐ No need for medications.<br>☐ Physical and occupational therapy are the main treatments.<br>☐ Medications are dangerous or harmful.<br>☐ Children do not get scleroderma.<br>☐ No, I was never told any of the above.<br>☐ Other
Please specify:	__________________________________
For systemic scleroderma, has a healthcare provider recommended treatment with methotrexate, mycophenolate mofetil (Cellcept), steroids, and/or other oral, injection, or intravenous (IV) medicines?	◯ Yes, my child was treated with one of these medications<br>◯ Yes, but we declined treatment with these medications<br>◯ No<br>◯ Unsure<br>◯ Prefer not to say
How much time passed between your child's first symptoms and starting systemic treatment (e.g., steroids, methotrexate, immunoglobulin, etc.)?	◯ Less than 3 months<br>◯ 3-6 months<br>◯ 6- 12 months<br>◯ 12-18 months<br>◯ 18-24 months<br>◯ 2-3 years<br>◯ 3-4 years<br>◯ More than 4 years
What made you decide to treat your child's scleroderma with medicine? Click all that apply	☐ My child's primary care provider agreed with this treatment<br>☐ I received information from the Scleroderma Foundation about this treatment<br>☐ I talked to another family whose child has scleroderma about the treatment<br>☐ I trusted the doctor who advised this treatment<br>☐ My child's disease was worsening<br>☐ Their current treatment was not working (please list what treatment they were on in "other")<br>☐ Other
Please specify:	__________________________________

What made you decide to decline medicines for your child's scleroderma? Click all that apply	☐ I was worried about the side effects and safety of these medicines<br>☐ I preferred to try natural treatments such as herbs, diet, or lifestyle changes<br>☐ I cannot afford the recommended treatments/medicines<br>☐ My child's scleroderma was mild<br>☐ Other
Please specify:	__________________________________
Has your child been referred to a surgeon for functional or appearance concerns?	◯ Yes<br>◯ No
What type of surgeon(s) have you seen?	__________________________________
Please feel free to provide any additional information regarding difficulties for obtaining medical care for your child's scleroderma:	____
